# Transitions of blood immune endotypes and improved outcome by anakinra in COVID-19 pneumonia: an analysis of the SAVE-MORE randomized controlled trial

**DOI:** 10.1186/s13054-024-04852-z

**Published:** 2024-03-12

**Authors:** Evdoxia Kyriazopoulou, Yehudit Hasin-Brumshtein, Uros Midic, Garyfallia Poulakou, Haralampos Milionis, Simeon Metallidis, Myrto Astriti, Archontoula Fragkou, Aggeliki Rapti, Eleonora Taddei, Ioannis Kalomenidis, Georgios Chrysos, Andrea Angheben, Ilias Kainis, Zoi Alexiou, Francesco Castelli, Francesco Saverio Serino, Petros Bakakos, Emanuele Nicastri, Vasiliki Tzavara, Sofia Ioannou, Lorenzo Dagna, Katerina Dimakou, Glykeria Tzatzagou, Maria Chini, Matteo Bassetti, Vasileios Kotsis, Dionysios G. Tsoukalas, Carlo Selmi, Alexandra Konstantinou, Michael Samarkos, Michael Doumas, Aikaterini Masgala, Konstantinos Pagkratis, Aikaterini Argyraki, Karolina Akinosoglou, Styliani Symbardi, Mihai G. Netea, Periklis Panagopoulos, George N. Dalekos, Oliver Liesenfeld, Timothy E. Sweeney, Purvesh Khatri, Evangelos J. Giamarellos-Bourboulis

**Affiliations:** 1 4th Department of Internal Medicine, Medical School, National and Kapodistrian University of Athens, Athens, Greece; 2 Inflammatix, Inc., Sunnyvale, CA USA; 3 3rd Department of Internal Medicine, Medical School, National and Kapodistrian University of Athens, Athens, Greece; 4 1st Department of Internal Medicine, Medical School, University of Ioannina, Ioannina, Greece; 5 1st Department of Internal Medicine, Medical School, Aristotle University of Thessaloniki, Thessaloniki, Greece; 6 1st Department of Internal Medicine, G. Gennimatas General Hospital of Athens, Athens, Greece; 7 Department of Internal Medicine, Elpis General Hospital, Athens, Greece; 8 2nd Department of Pulmonary Medicine, Sotiria General Hospital of Chest Diseases, Athens, Greece; 9 Dipartimento Scienze di Laboratorio e Infettivologiche, Fondazione Policlinico Gemelli IRCCS, Rome, Italy; 10 1st Department of Critical Care and Pulmonary Medicine, Medical School, National and Kapodistrian University of Athens, Evangelismos General Hospital, Athens, Greece; 11 2nd Department of Internal Medicine, Tzaneio General Hospital of Piraeus, Athens, Greece; 12 Department of Infectious – Tropical Diseases and Microbiology, IRCSS Sacro Cuore Hospital, Negrar, Verona, Italy; 13 10th Department of Pulmonary Medicine, Sotiria General Hospital of Chest Diseases of Athens, Athens, Greece; 14 2nd Department of Internal Medicine, Thriasio General Hospital of Eleusis, Athens, Greece; 15 Spedali Civili, Brescia ASST Spedali Civili Hospital, University of Brescia, Brescia, Italy; 16 Department of Internal Medicine, Hospital of Jesolo, Jesolo, Italy; 17 1st Department of Chest Medicine, Medical School, National and Kapodistrian University of Athens, Athens, Greece; 18 Department of Internal Medicine, Spallanzani Institute of Rome, Rome, Italy; 19 1st Department of Internal Medicine, Korgialeneion-Benakeion General Hospital, Athens, Greece; 20 Department of Therapeutics, Medical School, National and Kapodistrian University of Athens, Athens, Greece; 21 Unit of Immunology, Rheumatology, Allergy and Rare Diseases (UnIRAR), IRCCS Ospedale San Raffaele and Vita-Salute San Raffaele University, Milan, Italy; 22 5th Department of Pulmonary Medicine, Sotiria General Hospital of Chest Diseases, Athens, Athens, Greece; 23 1st Department of Internal Medicine, Papageorgiou General Hospital of Thessaloniki, Thessaloniki, Greece; 24 3rd Department of Internal Medicine and Infectious Diseases Unit, Korgialeneion-Benakeion General Hospital, Athens, Greece; 25 Infectious Diseases Clinic, Ospedale Policlinico San Martino IRCCS and Department of Health Sciences, University of Genova, Genova, Italy; 26 3rd Department of Internal Medicine, Medical School, Aristotle University of Thessaloniki, Thessaloniki, Greece; 27 4th Department of Pulmonary Medicine, Sotiria General Hospital of Chest Diseases, Athens, Greece; 28 Department of Biomedical Sciences, Humanitas University and IRCCS Humanitas Research Hospital, Milan, Italy; 29 1st Department of Internal Medicine, Asklepieio General Hospital of Voula, Voula, Greece; 30 1st Department of Internal Medicine, Medical School, National and Kapodistrian University of Athens, Athens, Greece; 31 2nd Department of Propedeutic Medicine, Medical School, Aristotle University of Thessaloniki, Thessaloniki, Greece; 32 2nd Department of Internal Medicine, Konstantopouleio General Hospital, Athens, Greece; 33 Department of Pulmonary Medicine, General Hospital of Kerkyra, Corfu, Greece; 34 Department of Internal Medicine, Sotiria General Hospital of Chest Diseases, Athens, Greece; 35 Department of Internal Medicine, University of Patras, Patras, Greece; 36 1st Department of Internal Medicine, Thriasio General Hospital of Eleusis, Athens, Greece; 37 Department of Internal Medicine and Center for Infectious Diseases, Radboud University, Nijmegen, The Netherlands; 38 Department of Immunology and Metabolism, Life and Medical Sciences Institute, University of Bonn, Bonn, Germany; 39 2nd Department of Internal Medicine, Medical School, Democritus University of Thrace, Alexandroupolis, Greece; 40 Department of Medicine and Research Laboratory of Internal Medicine, National Expertise Center of Greece in Autoimmune Liver Diseases, European Reference Network on Hepatological Diseases (ERN RARE-LIVER), General University Hospital of Larissa, Larissa, Greece; 41 Institute for Immunity, Transplantation and Infection, School of Medicine, Stanford University, Stanford, CA USA; 42 Center for Biomedical Informatics Research, Department of Medicine, Stanford University, Stanford, CA USA; 43 4th Department of Internal Medicine, ATTIKON University Hospital, 1 Rimini Street, 124 62, Athens, Greece

**Keywords:** Endotypes, Anakinra, COVID-19, Viral sepsis

## Abstract

**Background:**

Endotype classification may guide immunomodulatory management of patients with bacterial and viral sepsis. We aimed to identify immune endotypes and transitions associated with response to anakinra (human interleukin 1 receptor antagonist) in participants in the SAVE-MORE trial.

**Methods:**

Adult patients hospitalized with radiological findings of PCR-confirmed severe pneumonia caused by SARS-CoV-2 and plasma-soluble urokinase plasminogen activator receptor levels of ≥ 6 ng/ml in the SAVE-MORE trial (NCT04680949) were characterized at baseline and days 4 and 7 of treatment using a previously defined 33-messenger RNA classifier to assign an immunological endotype in blood. Endpoints were changes in endotypes and progression to severe respiratory failure (SRF) associated with anakinra treatment.

**Results:**

At baseline, 23.2% of 393 patients were designated as inflammopathic, 41.1% as adaptive, and 35.7% as coagulopathic. Only 23.9% were designated as the same endotype at days 4 and 7 compared to baseline, while all other patients transitioned between endotypes. Anakinra-treated patients were more likely to remain in the adaptive endotype during 7-day treatment (24.4% vs. 9.9%; *p* < 0.001). Anakinra also protected patients with coagulopathic endotype at day 7 against SRF compared to placebo (27.8% vs. 55.9%; *p* = 0.013).

**Conclusion:**

We identify an association between endotypes defined using blood transcriptome and anakinra therapy for COVID-19 pneumonia, with anakinra-treated patients shifting toward endotypes associated with a better outcome, mainly the adaptive endotype.

*Trial registration* ClinicalTrials.gov, NCT04680949, December 23, 2020.

## Background

Sepsis, defined as a dysregulated host immune response to infection resulting in life-threatening organ dysfunction, is one of the leading causes of death, affecting as many as 50 million individuals annually with mortality as high as 40% [1, 2]. Coronavirus 2019 disease (COVID-19) that rapidly turned to a pandemic spreading around the globe and leading to millions of confirmed cases and deaths worldwide is characterized by complex immune dysregulation; severe pneumonia is often associated with acute dysfunction of additional organs such as the kidney or the circulation [3–5]. Consequently, the Sepsis-3 definition may apply for COVID-19. Indeed, analysis applying the Sepsis-3 criteria showed that almost 80% of COVID-19 patients hospitalized in the intensive care unit (ICU) met the criteria, and they can be considered as sufferers from viral sepsis [6, 7].

Sepsis is a heterogeneous syndrome, and many investigators have introduced the need of subgroup classification into endotypes reflecting the mechanism of the disease [8]. Patient stratification subsequently allows the tailoring of precision immunotherapy. These endotypes share common immunobiological pathways and may guide targeted immunomodulatory treatment [9].

Recently, a 33-gene-based classifier for patient stratification in sepsis has been developed. The classifier assigns a patient with sepsis to one of three distinct endotypes: inflammopathic, adaptive and coagulopathic [10]. The combination of the expression of *ARG1, LCN2, LTF, OLFM4, HLA-DMB* defines the inflammopathic endotype; of *YKT6, PDE4B, TWISTNB, BTN2A2, ZBTB33, PSMB9, CAMK4, TMEM19, SLC12A7, TP53BP1, PLEKHO1, SLC25A22, FRS2, GADD45A, CD24, S100A12, STX1A* the adaptive endotype; and of *KCNMB4, CRISP2, HTRA1, PPL, RHBDF2, ZCCHC4, YKT6, DDX6, SENP5, RAPGEF1, DTX2, RELB* the coagulopathic endotype. These endotypes were validated in an independent cohort of patients with COVID-19 pneumonia at the beginning of the pandemic. Validation showed that that patients of the inflammopathic endotype had the highest circulating concentrations of C-reactive protein (CRP) and those of the coagulopathic endotype the highest circulating concentrations of D-dimers. Patients of the adaptive endotype had the best 28-day outcome [11].

SAVE-MORE is a pivotal randomized clinical trial where patients with COVID-19 pneumonia at early activation of the interleukin (IL)-1 cascade recognized by increased blood concentrations of the biomarker suPAR (soluble urokinase plasminogen activator receptor) were randomized to treatment with placebo or anakinra. Results showed that anakinra treatment was accompanied by an improved outcome compared to placebo by day 28, as expressed by the WHO Clinical Progression Scale (WHO-CPS) [12]. Based on this evidence, anakinra was approved for the treatment of COVID-19 pneumonia by both the European Medicines Agency and by the Food and Drug Administration of the US [13, 14]. We applied the 33-messenger RNA (mRNA) endotype classifier to SAVE-MORE participants to identify which sepsis immune endotypes are the best candidates for anakinra treatment.

## Methods

### Patients

This study is a *post hoc* analysis of the SAVE-MORE double-blind randomized clinical trial (NCT04680949), approved by the National Ethics Committee of Greece (approval 161/20) and by the Ethics Committee of the National Institute for Infectious Diseases Lazzaro Spallanzani, IRCCS, in Rome (1 February 2021) [11]. In SAVE-MORE, trial participants were 1:2 randomly allocated to once daily subcutaneous treatment with either placebo or anakinra 100 mg for 10 days in addition to Standard-of-Care (SoC). Dexamethasone, remdesivir and anticoagulation were allowed in the SoC at the discretion of treating physicians; other anti-cytokine drugs like tocilizumab were not allowed. Study participants were adults of either sex, hospitalized with radiological findings of pneumonia by SARS-CoV-2, and plasma suPAR 6 ng/ml or more. Infection was confirmed by PCR testing. Main exclusion criteria were: noninvasive or invasive mechanical ventilation, stage IV malignancy, any do-not-resuscitate decision, ratio of partial oxygen pressure to fraction of inspired oxygen less than 150, severe hepatic failure, any primary immunodeficiency, neutrophils less than 1500/mm^3^, oral or intravenous corticosteroids more than 0.4 mg/kg/day of equivalent prednisone the last 15 days, any anti-cytokine biologic treatment the last month, hemodialysis, and pregnancy or lactation. All patients or their legal representatives provided written informed consent before enrollment. The current analysis included only participants with severe disease at baseline according to the WHO definition (i.e., respiratory rate > 30 breaths/min, severe respiratory distress, or SpO_2_ < 90% on room air) providing at least a baseline blood sample [15].

### RNA extraction and NanoString profiling

RNA extraction was performed from 2-ml aliquot of the available sample with RNeasy Micro Plus kit (Qiagen, Cat. No. / ID: 74134) according to manufacturer protocol. Elution volume was 14 µl, with average total yield of 720 ng. Each NanoString expression profiling reaction consisted of 150 ng of RNA per sample hybridized for 16 h at 65 °C per manufacturer’s instructions. We then followed the nCounter SPRINT standard protocol to generate mRNA counts.

We normalized mRNA counts using geometric mean of counts of four housekeeping genes (*CDIPT, KPNA6, RREB1* and *YWHAB*) to account for differences in hybridization, purification, binding efficiency, and other experimental variables as described in nCounter^®^ Expression Data Analysis Guide (https://nanostring.com/wp-content/uploads/Gene_Expression_Data_Analysis_Guidelines.pdf). To assess sample quality, we used geometric mean of housekeeping gene counts (pre-normalization) and limit-of-detection (LOD).

### Laboratory procedures and endotypes

Whole blood was drawn in PAXgene RNA tubes (Becton Dickinson) at three timepoints (before start of the study drug and days 4 and 7 of treatment), along with other standard laboratory parameters. PAXgene blood RNA samples were shipped to Inflammatix, where RNA was extracted and the 33 mRNAs were quantitated using NanoString nCounter (NanoString, Seattle, WA). Endotypes were grouped as previously described [9]. Briefly, each of the 33 mRNAs is assigned to one of the three groups, and the difference of geometric means of gene expression for each grouping is calculated. The previously defined multiclass logistic regression model was applied to these three input gene expression scores, which yields a probability of endotype assignment (for each subject, the total probability (p[Inflammopathic] + p[Adaptive] + p[Coagulopathic] sums to 1). Each sample is assigned an endotype according to the highest probability.

### Endpoint

This analysis is a secondary endpoint of the study protocol which received advice from the Emergency Task Force for COVID-19 of the European Medicines Agency (document EMA/659928/2020). The analysis identified endotype transitions which may be associated with anakinra treatment and the association of endotypes or endotype transitions from baseline to day 7 of treatment with the progression into severe respiratory failure (SRF) and/or death at day 28. SRF was defined as the need for noninvasive or invasive mechanical ventilation at day 28.

### Statistical analysis

Categorical data were presented as frequencies and confidence intervals (CI); continuous variables with normal distribution as mean with standard deviation (SD). Fisher’s exact/Chi-square test was used for comparison of categorical data, whereas Student’s t test/ANOVA or nonparametric Mann–Whitney/Kruskal–Wallis tests were used for the comparison of continuous data, as appropriate. Odds ratio (OR) with CI was calculated for categorical data. Cox regression analysis was used to detect the impact of endotype transitions on clinical outcomes at day 28; hazard ratio (HR) and 95% CI were calculated. In order to investigate if stabilization of patients to specific endotypes is not influenced by confounding factors like age, comorbidities, suPAR levels, COVID-19 severity, treatment with dexamethasone, body mass index and country, univariate and multivariate logistic regression analyses were done. In the model stabilization to a specific endotype, over the three timepoints of sampling was the dependent variable, and all confounding factors and anakinra treatment were the independent variables. Quantitative variables entered the model as binary variables pre-defined by the Emergency Task Force of the European Medicines Agency for COVID-19 as follows [16]: (a) age ≥ 65 years or less than 65 years; (b) Charlon’s comorbidity index (CCI) ≥ 2 or less than 2; (c) suPAR ≥ 9 ng/ml or less than 9 ng/ml; and d) Sequential Organ Failure Assessment (SOFA) score ≥ 3 or less than 3. COVID-19 severity, treatment with dexamethasone, body mass index and country were the randomization strata of the SAVE-MORE trial. Any two-sided *p* value < 0.05 was considered statistically significant. Statistical analysis was performed using the software SPSS version 29.0.

## Results

### Patients

In the original trial, 594 patients were enrolled: 189 were treated with SoC and placebo, and 405 were treated with SoC and anakinra. In the present analysis, 393 patients with severe pneumonia providing at least a baseline blood sample, were included, of which 130 patients were allocated to treatment with SoC and placebo and 263 patients to treatment with SoC and anakinra. Characteristics of anakinra- and placebo-treated patients did not differ at baseline (Table 1). Blood samples were available from all patients at baseline, 327 patients at day 4 of treatment; and 326 patients at day 7 of treatment. For 282 patients, blood samples were available at all three timepoints. At baseline, 101 patients (25.7%) were classified in the inflammopathic endotype, 148 patients (37.7%) in the adaptive endotype, and 144 patients (36.6%) in the coagulopathic endotype. Patients with inflammopathic or coagulopathic endotype had higher sequential organ failure assessment (SOFA) score, more respiratory distress (as attested by a lower respiratory ratio) and higher inflammatory burden (as attested by the higher white blood cell count and the higher levels of CRP and of ferritin) when compared to patients with the adaptive endotype (Table 2).

**Table 1:** Characteristics of patients by group of treatment

	Placebo + SoC (*N* = 130)	Anakinra + SoC (*Ν* = 263)	*p*
Age, years, mean (SD)	62 (12)	62 (12)	0.626
Male sex, *n* (%)	78 (60.0)	154 (58.6)	0.828
Body mass index, mean (SD)	30.2 (5.9)	29.7 (5.6)	0.321
Charlson’s comorbidity index, mean (SD)	2.2 (1.5)	2.2 (1.6)	0.543
SOFA score, mean (SD)	2.5 (1.1)	2.6 (1.0)	0.958
*Comorbidities, n (%)*			
Type 2 diabetes mellitus	20 (15.4)	40 (15.2)	1.000
Chronic heart failure	2 (1.5)	8 (3.0)	0.507
Chronic renal disease	1 (0.8)	4 (1.5)	1.000
Chronic obstructive pulmonary disease	5 (3.8)	12 (4.6)	1.000
*Co-administered medications, n (%)*			
Remdesivir	92 (70.8)	192 (73.0)	0.634
Dexamethasone	128 (98.5)	259 (98.5)	1.000
Prophylactic low molecular weight heparin	78 (97.5)	175 (98.9)	0.591
*Outcomes, n (%)*			
Incidence of SRF at day 28	47 (36.2)	68 (25.9)	**0.045**
28-Day mortality	12 (9.3)	10 (3.8)	**0.035**

*P*-values of statistical significance are marked in bold

*SD* standard deviation, *SoC* standard-of-care, *SOFA* sequential organ failure assessment, *SRF* severe respiratory failure

**Table 2:** Characteristics of all patients by endotype assignment at day 1

	All patients (*N* = 393)	Inflammopathic (*N* = 101)	Adaptive (*Ν* = 148)	Coagulopathic (*Ν* = 144)	*p*
Age, years, mean (SD)	62 (12)	62 (12)	61 (12)	63 (12)	0.477
Male sex, *n* (%)	232 (59.0)	62 (61.4)	83 (56.1)	87 (60.4)	0.645
Body mass index, mean (SD)	29.9 (5.6)	29.2 (5.5)	30.7 (5.6)	29.6 (5.8)	0.058
Charlson’s comorbidity index, mean (SD)	2.2 (1.5)	2.3 (1.6)	2.1 (1.4)	2.3 (1.7)	0.671
SOFA score, mean (SD)	2.6 (1.1)	2.7 (1.2)	2.3 (1.0)*^,#^	2.7 (1.0)	**0.006**
*Comorbidities, n (%)*					
Type 2 diabetes mellitus	60 (15.3)	12 (11.9)	25 (16.9)	23 (16.0)	0.535
Chronic heart failure	10 (2.5)	3 (3.0)	4 (2.7)	3 (2.1)	0.899
Chronic renal disease	5 (1.3)	0 (0.0)	1 (0.7)	4 (2.8)	0.115
Chronic obstructive pulmonary disease	17 (4.3)	5 (5.0)	9 (6.1)	3 (2.1)	0.229
*Co-administered medications, n (%)*					
Assigned to Anakinra intervention	263 (66.9)	61 (60.4)	108 (73.0)	94 (65.3)	0.102
Remdesivir	284 (72.3)	67 (66.3)	119 (80.4)	98 (68.1)	**0.019**
Dexamethasone	387 (98.5)	100 (99.0)	146 (98.6)	141 (97.9)	0.771
Low molecular weight heparin	253 (64.4)	59 (58.4)	104 (70.3)	90 (62.5)	0.262
Venous thromboembolic event, *n* (%)	8 (2.0)	3 (2.9)	2 (1.3)	3 (2.1)	0.673
Arterial thrombosis, *n* (%)	1 (0.3)	0 (0.0)	0 (0.0)	1 (0.7)	0.420
*Laboratory values, median (Q1–Q3)*					
White blood cells, /mm^3^	6380 (4570–8715)	7370 (5470–9640)	5390 (4190–7180)*^,#^	6880 (4670–9465)	** < 0.0001**
Lymphocytes, /mm^3^	770 (560–1050)	650 (500–900)	980 (710–1185)*^,#^	670 (520–900)	** < 0.0001**
Platelets, /mm^3^	208,000 (164,000–274500)	210,000 (175,000–273000)	186,000 (154,500–243000)*^,#^	226,000 (171,000–298500)	**0.007**
C-reactive protein, mg/l	52.0 (25.6–102.4)	72.8 (25.4–114.6)	40.4 (21.4–76.8)*^,#^	63.0 (31.3–112.3)	**0.003**
Interleukin-6, pg/ml	16.3 (6.4–40.9)	15.1 (5.8–33.1)	19.6 (7.2–45.7)	16.0 (6.1–43.8)	0.061
Ferritin, ng/ml	635.9 (351.0–1146.4)	860.5 (446.0–1520.1)	533.6 (294.9–934.5)*	674.0 (310.6–1099.6)^$^	** < 0.0001**
D-dimers, mg/l	0.53 (0.32–0.95)	0.59 (0.33–1.16)	0.46 (0.28–0.77)*^,#^	0.55 (0.37–0.92)	**0.040**
Serum soluble uPAR, ng/ml	7.9 (7.0–8.9)	7.7 (6.8–9.5)	8.0 (7.0–8.5)	8.0 (6.8–8.9)	0.835
PaO_2_: FiO2	216 (172–275)	203 (154–250)	246 (199–293)*^,#^	203 (159–274)	** < 0.0001**

*P*-values of statistical significance are marked in bold

*SD* standard deviation, *SOFA* sequential organ failure assessment, *Q* quartile, *uPAR* urokinase plasminogen activator receptor

*p < 0.05 for comparison between adaptive and inflammopathic endotype

^*#*^*p* < 0.05 for comparison between adaptive and coagulopathic endotype

^$^*p* < 0.05 for comparison between inflammopathic and coagulopathic endotype

### Endotype assignment at baseline, days 4 and 7

**Fig. 1:**
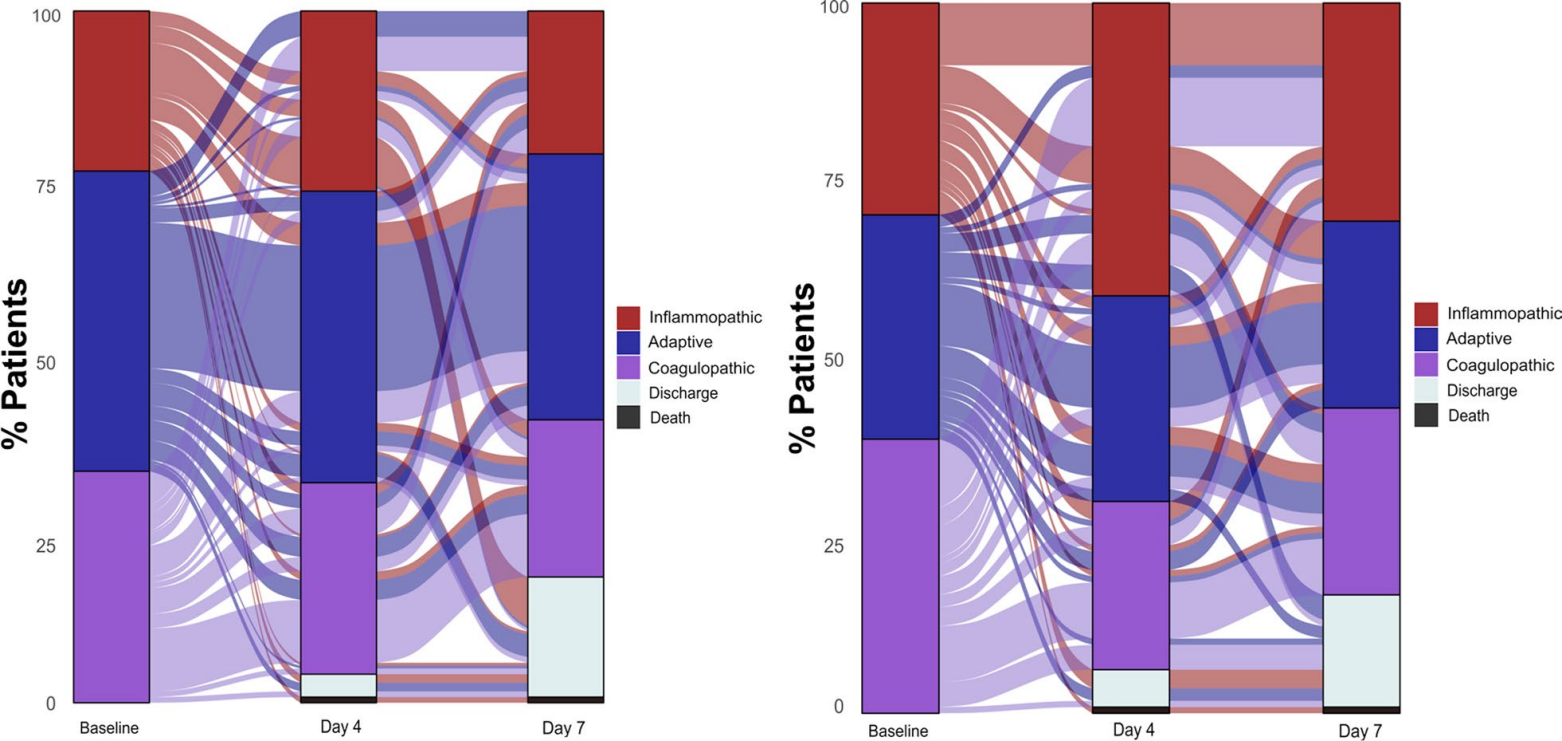
Alluvial plots of distribution of endotype transitions of patients of the SAVE-MORE trial treated with standard-of-care (SoC) and anakinra (*left panel*) and standard-of-care (SoC) and placebo (*right panel*), from baseline endotype to the endotype of days 4 and 7 of treatment

At baseline (i.e., prior to starting the treatment), the distribution of the three endotypes was similar in anakinra-treated and placebo-treated patients. Among anakinra-treated patients, 23.2% were classified as inflammopathic, 41.1% as adaptive and 35.7% as coagulopathic and among placebo-treated patients 30.8%, 30.8% and 38.5%, respectively (*p*: 0.102, Fig. 1). At day 4 of treatment, 29.9% of anakinra-treated patients were inflammopathic, 40.7% adaptive and 29.4% coagulopathic compared to 44.3%, 30.2% and 25.5% of placebo-treated patients, respectively (*p*: 0.032). At day 7 of treatment, 31.8% of anakinra-treated patients were inflammopathic, 43.6% adaptive and 24.5% coagulopathic compared to 34.0%, 34.0% and 32.1% of placebo-treated patients, respectively (*p*: 0.196, Fig. 1).

### Endotype transitions are common and Adaptive endotype is protective

**Fig. 2:**
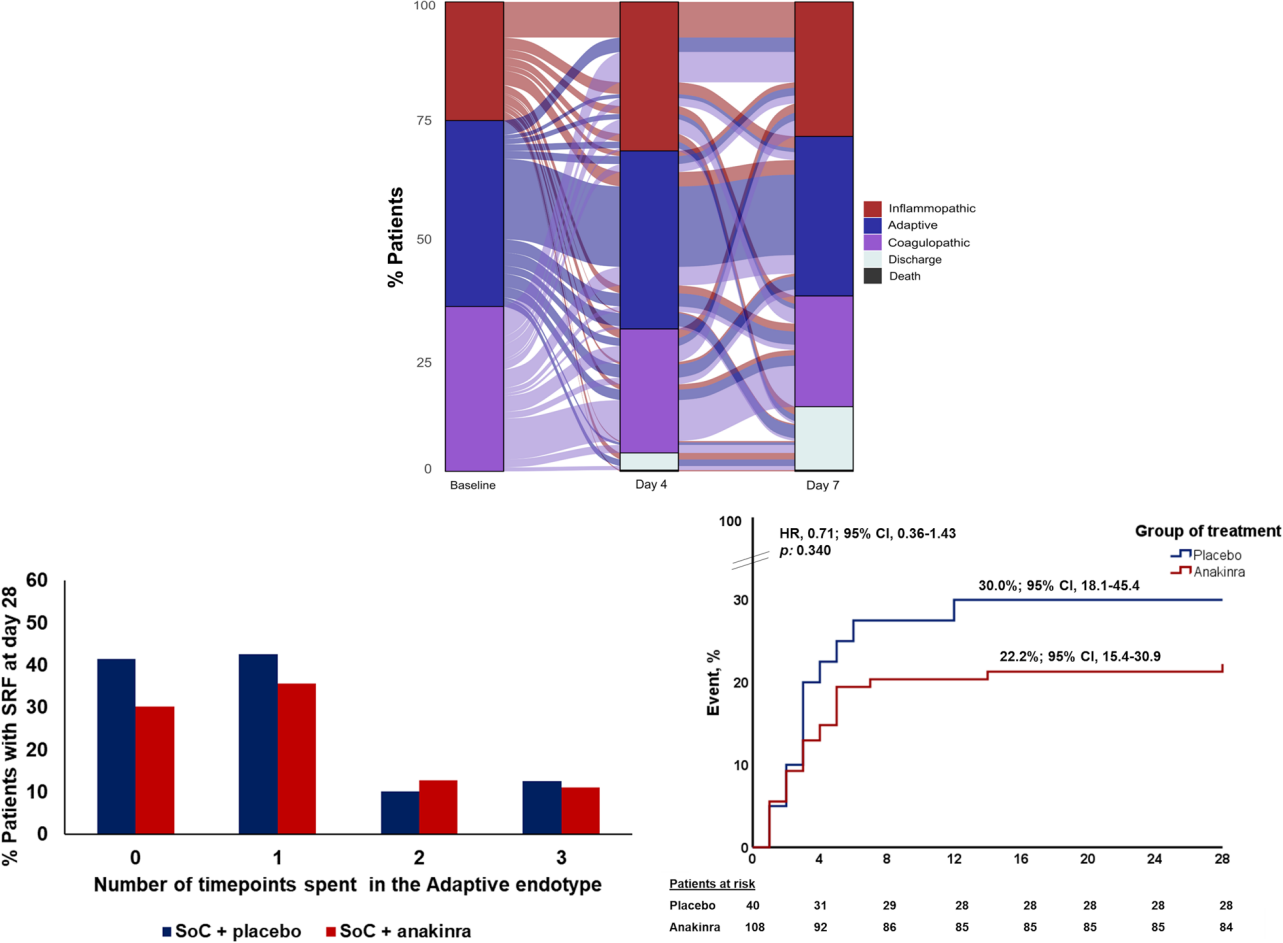
Endotype transitions between days of follow-up. *Upper panel:* Alluvial plot of distribution of endotype transitions of patients of the SAVE-MORE trial from baseline endotype to the endotype at days 4 and 7 of treatment. *Lower panel left:* Proportion of patients that develop severe respiratory failure (SRF) and/or die by day 28, as function of number of timepoints they spent in the adaptive endotype. *Lower panel right*: Kaplan–Meier curves for the time of progression to severe respiratory failure and/or death by day 28 of patients with adaptive endotype at baseline and treated with Standard-of-Care (SoC) and placebo or SoC and anakinra. Abbreviations: CI, confidence interval; HR, hazard ratio

Most patients changed endotypes over the three different timepoints; only 170 patients maintained the same endotype at day 4 as the baseline, whereas 94 patients maintained at day 7 the same endotype as both the baseline and day 4. For all other patients, an endotype transition was observed under treatment (Fig. 2). Adaptive endotype was associated with better outcomes irrespective of treatment arm. Among 371 patients with available serial data of at least two consecutive timepoints, 148 (37.7%) patients were never assigned an adaptive endotype, 96 (24.4%) were assigned an adaptive endotype at one timepoint, 83 (21.1%) at two timepoints and 44 (11.2%) at all three timepoints. Incidence of SRF by day 28 was 34.5% for patients who were never assigned the adaptive endotype, 38.5% for those assigned adaptive endotype at one timepoint, 12.0% for those assigned adaptive endotype at two timepoints and 11.4% for those assigned adaptive endotype at all three timepoints (*p* < 0.001); 28-day mortality was 9.5%, 6.3%, 1.2% and 0% in these groups, respectively (*p*: 0.020). Results were significant even after adjusting for remdesivir treatment and anticoagulation (HR_adj_, 0.63; 95% CI 0.49–0.81; *p* < 0.0001 for incidence of SRF and HR_adj_, 0.31; 95% CI 0.13–0.74; *p*: 0.008 for 28-day mortality, respectively). Patients who were assigned adaptive endotype at 2 or more timepoints were less likely to develop SRF (11.8% vs. 36.1%, *p* < 0.0001) or die within 28 days (0.8% vs. 8.2%, *p*: 0.002) than those who were assigned adaptive endotype at 1 or no timepoint. Incidence of SRF was again significant even after adjusting for remdesivir treatment and anticoagulation (HR_adj_, 0.29; 95% CI 0.16–0.54; *p* < 0.0001). The protective effect of the adaptive endotype was consistent and replicated in both placebo and anakinra arms of treatment (*p*: 0.454 by Tarone’s test; Fig. 2). The incidence of SRF and/or death was similar in both arms of treatment among patients with baseline adaptive endotype (Fig. 2).

### Anakinra treatment stabilizes the Adaptive endotype

**Fig. 3:**
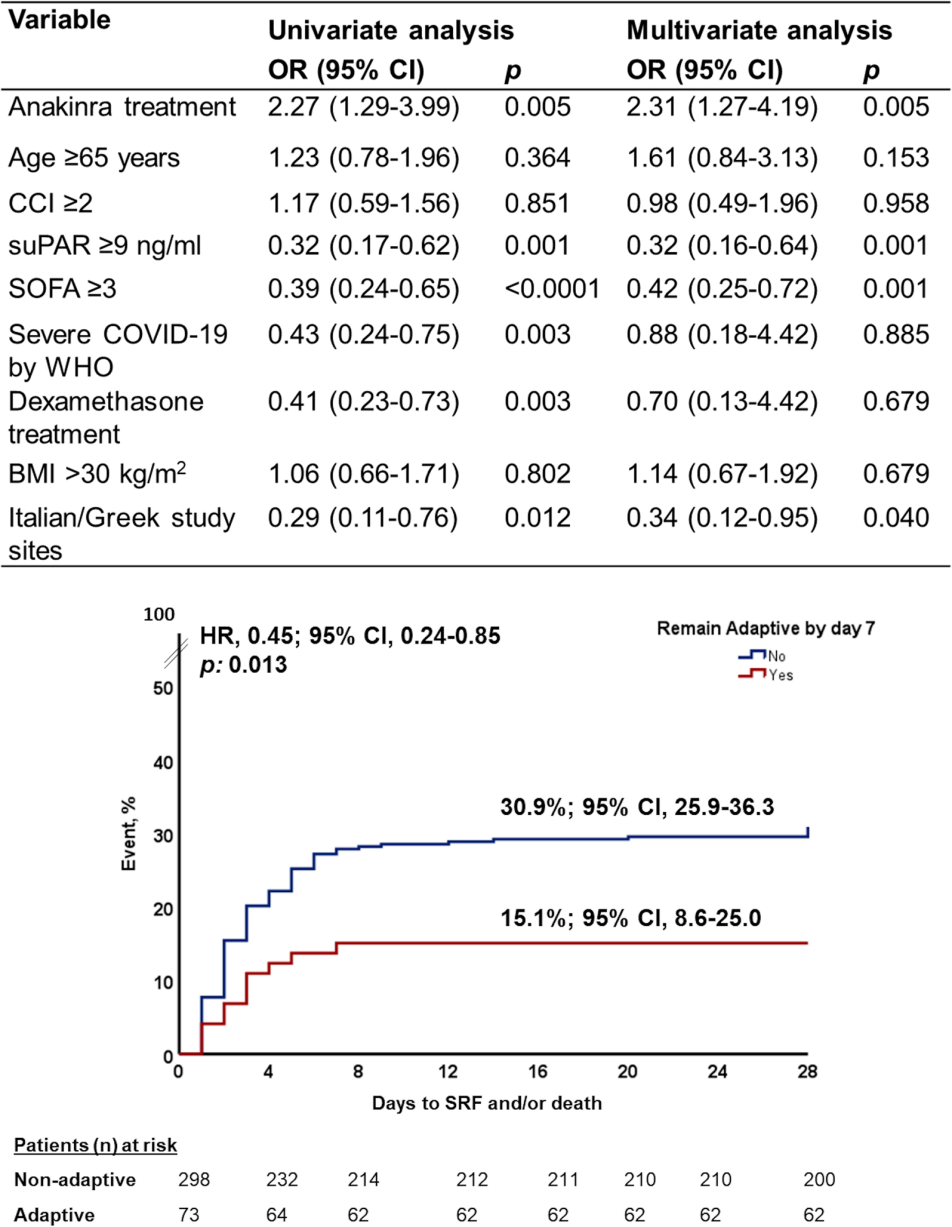
Stabilization over the first 7 days of follow-up to the adaptive endotype. The upper panel shows the univariate and multivariate logistic regression analyses of variables associated with the stabilization of patients in the adaptive endotype between baseline day 1 and follow-up days 4 and 7. The lower panel shows the Kaplan–Meier curves for the time of progression to severe respiratory failure and/or death by day 28 between patients of the entire SAVE-MORE cohort remaining by day 7 in the adaptive endotype and those not stabilized by day 7 in adaptive endotype. Abbreviations: BMI, body mass index; CCI, Charlson’s comorbidity index; CI, confidence interval; HR, hazard ratio; n, number; OR, odds ratio; SOFA, sequential organ failure assessment score; SRF, severe respiratory failure; suPAR, soluble urokinase plasminogen activator receptor

Anakinra treatment prevented shifting of patients from the adaptive to other endotypes under treatment compared to placebo (Fig. 1). More precisely, 24.4% of anakinra-treated patients were stabilized in the adaptive endotype compared to only 9.9% of placebo-treated patients (*p* < 0.0001). This effect was further demonstrated by multivariate logistic regression analysis taking into consideration all possible confounders. Univariate and multivariate analyses showed that anakinra treatment was the only independent variable favoring stabilization to the adaptive endotype, whereas disease severity as expressed by the level of suPAR and SOFA score was against stability into the adaptive endotype. Patients stabilized by day 7 to the adaptive endotype were at less risk for progression into SRF and/or death at day 28 (Fig. 3).

### Coagulopathic endotype at day 7 as predictor of final outcome

**Fig. 4:**
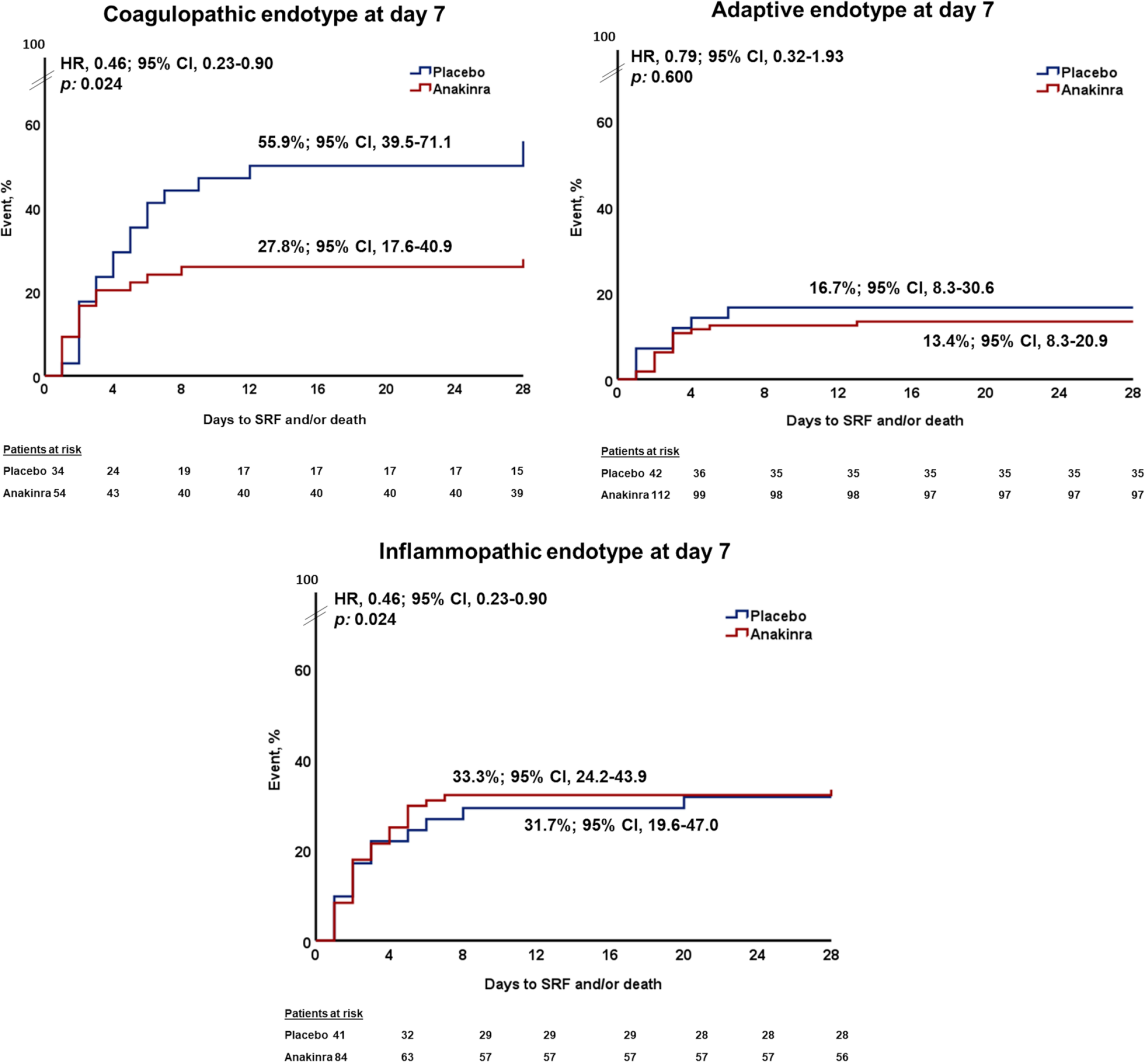
Association between the prevailing endotype at day 7 and 28-day outcome. Kaplan–Meier curves for the time of progression to severe respiratory failure and/or death by day 28 between patients treated with Standard-of-Care (SoC) and placebo versus Standard-of-Care (SoC) and anakinra and classified at day 7 into the coagulopathic endotype (*upper panel left*); the adaptive endotype (*upper panel right*); and the inflammopathic endotype (*lower panel*). Abbreviations: CI, confidence interval; HR, hazard ratio

Hyper-coagulation is a predominant feature of COVID-19 pneumonia associated with mortality [5]. Since the coagulopathic endotype is characterizing patients at a hyper-coagulable state [12], we analyzed the association between progression to SRF and incidence of the coagulopathic endotype at day 7. In detail, 34 patients allocated to the SoC and placebo arm had the coagulopathic endotype by day 7 (of these 34 patients, six patients were inflammopathic at baseline, 12 patients were adaptive at baseline and 16 patients were coagulopathic at baseline); 19 patients (55.9%) progressed to SRF. In total, 54 patients allocated to the SoC and anakinra arm had the coagulopathic endotype by day 7 (of these patients, 11 patients were inflammopathic at baseline, 14 patients were adaptive at baseline and 29 patients were coagulopathic at baseline); 15 patients (27.8%) progressed to SRF (*p*: 0.013 compared to the SoC and placebo arm), showing that anakinra treatment prevented the deleterious effect of the coagulopathic endotype associated with the development of SRF (Fig. 4). Anakinra-treated patients with coagulopathic endotype at day 7 had lower serum CRP, IL-6 and suPAR concentrations, and higher absolute lymphocyte count compared to placebo-treated patients with coagulopathic endotype at day 7 (Table 3). Incidence of SRF and/or death was similar between anakinra-treated patients and placebo-treated patients classified with adaptive or inflammopathic endotypes at day 7 (Fig. 4).

**Table 3:** Laboratory values of patients with coagulopathic endotype at day 7 by arm of treatment

Laboratory values, median (Q1–Q3)	SoC + Placebo (*Ν* = 34)	SoC + Anakinra (*Ν* = 54)	*p*
White blood cells, /mm^3^	8800 (7400 to 12,480)	8580 (6820 to 10,185)	0.684
Lymphocytes, /mm^3^	720 (535 to 1260)	1060 (735 to 1560)	**0.003**
Platelets, /mm^3^	314,000 (247,500 to 422,500)	363,000 (307,500 to 466,500)	**0.026**
C-reactive protein, mg/l	15.2 (6.8 to 51.6)	6.5 (2.5 to 27.9)	**0.001**
Interleukin-6, pg/ml	14.4 (3.4 to 59.2)	5.1 (1.4 to 14.7)	**0.016**
Ferritin, ng/ml	585.8 (355.9 to 889.0)	490.9 (247.2 to 825.8)	0.373
D-dimers, mg/l	0.72 (0.38 to 1.62)	0.81 (0.37 to 1.74)	0.910
Serum soluble uPAR, ng/ml	8.0 (6.3 to 12.1)	6.5 (4.7 to 8.4)	** < 0.001**
% Change of lymphocytes at day 7 from baseline	− 17.7 (− 71.8 to 13.3)	− 50.1 (− 97.5 to − 13.1)	**0.020**
% Change of C-reactive protein at day 7 from baseline	− 71.8 (− 90.9 to − 1.5)	− 91.9 (− 95.6 to − 67.2)	**0.002**
% Change of interleukin-6 at day 7 from baseline	− 16.8 (− 71.9 to 77.8)	− 49.1 (− 78.3 to 22.2)	0.136
% Change of ferritin at day 7 from baseline	− 8.0 (− 28.7 to 13.1)	− 32.8 (− 47.9 to − 10.4)	**0.010**
% Change of D-dimers at day 7 from baseline	32.1 (− 5.0 to 216.7)	6.6 (− 22.1 to 101.6)	0.208
% Change of suPAR at day 7 from baseline	20.3 (− 4.6 to 38.6)	− 17.4 (-43.2 to 9.1)	** < 0.0001**

*P*-values of statistical significance are marked in bold

*SoC* standard of care, *Q* quartile, *uPAR* urokinase plasminogen activator receptor

## Discussion

In this *post hoc* analysis of the SAVE-MORE clinical trial that randomized patients with COVID-19 pneumonia into SoC/placebo versus SoC/anakinra treatment arms, we show that hospitalized patients with severe COVID-19 pneumonia according to WHO definition are classified into three endotypes, namely inflammopathic, adaptive and coagulopathic. More than half of these patients change their immune endotype during disease course. Anakinra treatment stabilizes patients in the adaptive endotype, which is associated with a better outcome. Anakinra treatment also has a protective effect against the deleterious effect of the coagulopathic endotype.

Among the patients with COVID-19 admitted in the ICU, 80% fulfill the criteria of viral sepsis [7]. Sepsis in general is a heterogeneous clinical condition, and it is rational to hypothesize that similar heterogeneity applies to viral sepsis [17]. We previously described inflammopathic, adaptive and coagulopathic endotypes in patients with COVID-19 pneumonia [11], and the adaptive endotype was accompanied by the most favorable outcome. To the best of our knowledge, no other endotype classification has been validated in viral sepsis so far. Several other efforts have taken place to classify patients with bacterial sepsis into endotypes. Davenport et al. performed a genome-wide transcription profiling of patients admitted in the ICU due to community-acquired pneumonia and ended up with two sepsis response signature (SRS) groups, namely SRS1 and SRS2 [18]. Working toward the molecular diagnosis and risk stratification (MARS) of sepsis, Scicluna et al. defined four distinct endotypes, namely MARS 1–4 [19].

Endotypes may have a prognostic value; in various cohorts specific endotypes, such as the inflammopathic, the SRS1 and the MARS1 are associated with higher mortality [9]. The adaptive endotype as defined here was previously shown to be protective in COVID-19 patients, being associated with the lowest mortality [11]. Interestingly, remaining in the adaptive endotype throughout the first 7 days of follow-up is associated with decreased risk for SRF. In fact, almost all studies of endotypes so far describe the classification only at baseline and predict outcome only with the baseline time snapshot. We here show for the first time that more than 50% of patients evolve over time and are assigned to different endotypes. Moreover, endotype after 7 days of treatment is more predictive than baseline for unfavorable final outcomes. Thus, following longitudinally the endotype assignment of patients may be more important to detect alterations and possible effects of the applied treatment on the host immune response and clinical outcomes.

Anakinra treatment prevented shifting of the patients from the adaptive endotype. Adaptive endotype is characterized by activation of pathways of adaptive immune responses such as type I interferon antiviral response and this likely contributed to contain the virus but also deleterious immune responses, independently from antiviral treatment and anticoagulation [10]. Endotype classification was developed to detect suitable candidates for different immunomodulatory approaches, and with our results, this hypothesis seems to be valid; treating patients with anakinra and switching off the hyperinflammation reprograms the host’s immune function to a more adaptive setting protecting the host from SRF, multiorgan failure and death.

One striking finding of the current analysis is the importance of the coagulopathic endotype. Previous studies have shown that the coagulopathic endotype is associated with a deleterious outcome [10]. In this study, anakinra treatment protected these patients from developing SRF; the precise molecular pathway through which anakinra treatment exerts this beneficial effect needs to be studied further.

In a retrospective analysis of a subgroup of patients participating in the ORANGES trial who received hydrocortisone, ascorbic acid, and thiamine in sepsis, a first association was found between endotype (inflammopathic and coagulopathic), hydrocortisone and outcome [20]. In our cohort, almost all patients received dexamethasone for COVID-19 as SoC and such an association with corticosteroids was not feasible to detect.

It needs to be outscored that this if the first time where the association of an immunointervention with the over-time modulation of the host endotype is presented in a randomized clinical trial. The main strengths of the study are the randomized design, the use of validated endotypes and the serial measurements demonstrating endotype evolution over time. The main limitations are: (a) the lack of samples from all patients at all time points; (b) the enrollment of patients with increased suPAR; and (c) the existing, so far, difficulty of application of endotypes in daily routine practice. The investigation of the endotypes in immunocompromised and transplanted patients should become a future priority.

## Conclusions

To the best of our knowledge, this is the first study to assess the clinical utility of endotype classification at different timepoints in COVID-19 with clinical benefit of immunomodulatory treatment with anakinra. Patient stratification and a personalized approach of immunotherapy is likely to become a cornerstone for the future of sepsis management, and more research is needed toward this direction.

## Data Availability

All data are presented in tables and figures. Any supplementary data are available by the corresponding author upon reasonable request.

## Abbreviations

CI: Confidence intervals
CRP: C-reactive protein
HR: Hazard ratio
IL: Interleukin
ICU: Intensive Care Unit
MARS: Molecular diagnosis and risk stratification
mRNA: Messenger RNA
OR: Odds ratio
PCR: Polymerase chain reaction
SD: Standard deviation
SoC: Standard-of-care
SRF: Severe respiratory failure
SRS: Sepsis response signature suPAR: soluble urokinase plasminogen activator receptor
WHO-CPS: World Health Organization Clinical Progression Scale
